# Neutrophil lymphocyte ratio and duration of prior anti-angiogenic therapy as biomarkers in metastatic RCC receiving immune checkpoint inhibitor therapy

**DOI:** 10.1186/s40425-017-0287-5

**Published:** 2017-10-17

**Authors:** Ghayathri Jeyakumar, Seongho Kim, Naresh Bumma, Craig Landry, Cynthia Silski, Stacey Suisham, Brenda Dickow, Elisabeth Heath, Joseph Fontana, Ulka Vaishampayan

**Affiliations:** 1 Department of Oncology, Karmanos Cancer Institute/Wayne State University, 4 HWCRC 4100 John R, Detroit, MI 48201 USA; 2 Biostatistics Core, Karmanos Cancer Institute, Department of Oncology, School of Medicine, Wayne State University, Detroit, MI USA

**Keywords:** Biomarkers, Kidney cancer, Predictive, Prognostic marker, Neutrophil lymphocyte ratio, Immune checkpoint inhibitors

## Abstract

**Background:**

There is an unmet need to determine factors predictive of clinical benefit, to guide therapeutic sequencing and selection in metastatic RCC (mRCC). We evaluated clinical factors such as the neutrophil lymphocyte ratio (NLR) and duration of prior anti-vascular endothelial growth factor (VEGF) inhibitors, as predictors of response rate, progression free survival (PFS) and overall survival (OS) in mRCC patients treated with immune checkpoint inhibitor (ICI).

**Methods:**

Regulatory approval was obtained. A single center retrospective chart review of mRCC patients at Karmanos Cancer Institute, treated with ICI based therapy (PD-1/PD-L1 inhibitors) was conducted. Data were collected on demographics, smoking status, prognostic scoring (Memorial Sloan Kettering and Heng criteria), NLR pretherapy, post 1 and 4 doses of ICI, and duration of prior anti-VEGF therapy ≥6 months or <6.

**Results:**

42 patients were evaluated with median age of 61 years (range, 24-85). Pretherapy NLR < 3 and ≥3 was seen in 19 (45%) and 23 (55%) patients, respectively. 24 (57%) and 18 (43%) patients had prior anti-VEGF inhibitors for a duration of ≥6 months and <6 months, respectively. 12 (29%), 22 (52%) and 8 (19%) patients had favorable, intermediate and poor risk disease based on Heng criteria, respectively. Multivariable analysis showed pretherapy NLR ≥3 was predictive of shorter PFS and OS when treated with ICI with median 3.08 months and 13.50 months, respectively, versus 15.57 months and not reached for NLR < 3 (adjusted *p*-values =0.003 and 0.025, respectively). Prior anti-VEGF therapy <6 months was predictive of increased likelihood of benefit from ICI therapies (adjusted *p* = 0.028). The median PFS was 3.72 months and 14.33 months, respectively, in cases with prior anti-VEGF therapy for ≥6 months and <6 months.

**Conclusion:**

Pretherapy NLR <3 and duration of prior anti-VEGF therapy of <6 months, are independent statistically significant predictors of longer PFS and OS with ICI therapy in mRCC. Validation is required in a larger sample size with multi-institutional collaboration.

**Electronic supplementary material:**

The online version of this article (10.1186/s40425-017-0287-5) contains supplementary material, which is available to authorized users.

## Background

Immune checkpoint inhibitor (ICI) therapy has a unique mechanism of action of restoring T cell mediated immune response by blocking PD-1 and PD L1 interaction [1]. This therapy has recently entered the therapeutic realm of metastatic renal cell cancer (mRCC). Nivolumab was initially evaluated in mRCC and showed a promising overall response rate (ORR) of 27% and progression-free survival (PFS) 56% at 24 weeks [2]. In a phase II trial of 168 patients who were treated with different doses (0.3 mg/kg, 2 mg/kg and 10 mg/kg) ORR were 20%, 22% and 20% respecitvely with median overall survival (OS) of 18.2 mths, 25.5 mths, and 24.7 mths respectively [1]. The trial that led to the FDA approval of nivolumab was a randomized study comparing nivolumab to standard oral everolimus therapy in RCC patents that were pretreated with at least one prior anti-VEGF TKI therapy. The primary endpoint was overall survival (OS). Nivolumab showed a statistically significant improvement in OS over everolimus with median OS of 25 months and 19.6 months respectively in a 821 patient study (*p* = 0.003) [3]. In a parallel randomized trial, cabozantinib demonstrated both progression free survival and OS benefit over everolimus and also received FDA approval [4]. Besides this two other therapies, axitinib and combination of lenvatinib and everolimus have also demonstrated progression free survival benefit and are approved in mRCC post anti-VEGF TKI therapy. With multiple options of therapy emerging in the second line setting, the sequencing of agents has become a significant challenge [5, 6]. There is an unmet need for utilization of biomarkers to help guide therapeutic selection in mRCC. This will clearly aid in optimization of existing therapies and prevent exposure to adverse effects of unnecessary therapies with minimal likelihood of clinical benefit. Predictive biomarkers will also help streamline the cost of therapies in RCC.

Memorial Sloan Kettering (MSKCC) and Heng criteria have been widely accepted and are utilized as prognostic models for metastatic renal cell carcinoma [7–9]. The former was reported in the setting of interferon therapy and the latter in the anti-VEGF therapy era. Although some factors such as time interval from nephrectomy to metastatic disease, hemoglobin, LDH, performance status and corrected calcium, are common to both, the Heng criteria added the inclusion of neutrophilia and thrombocytosis that portend a worse prognosis. With the approval of ICI therapy in mRCC and the availability of multiple other systemic therapy choices, there is an unmet need for updates to the prognostic characteristics. In mRCC, the search for predictive markers to guide therapeutic selection has been disappointing to date. Multiple studies report no association of predictive biomarkers that have been evaluated in conjunction with sunitinib, pazopanib and everolimus therapies. For cabozantinib therapy cMET expression was evaluated and did not result in predictive impact [4].

Cancer-related inflammation has been reported to be a marker of poor prognosis [10]. Systemic inflammatory markers such as C-reactive protein have been used to make prognostic determinations of clinical outcome in different types of cancers. There are prior studies on neutrophil lymphocyte ratio (NLR) as a predictive marker in mRCC. Baum et al. [11] described that a NLR ≥4 was likely to be associated with a shorter OS as compared to patients with NLR <4. Other publications have shown better prognosis with lower NLR in the setting of therapies such as cytokines like interleukin-2 and anti-VEGF therapy such as sunitinib [12]. Hu et al. performed a meta-analysis to understand the prognostic value of NLR in RCC [10]. The study demonstrated that higher NLR is likely to be associated with shorter OS. A NLR cutoff of 3 was determined by conducting this meta-analysis combining data from 15 cohorts that showed increased risk of death (HR of 1.82;95% CI, 1.51 to 2.19) in mRCC cases with NLR ≥3.

We evaluated the potential role of clinical factors such as NLR and duration of benefit from prior anti-vascular endothelial growth factor (VEGF) inhibitors, as predictors of response rates, progression free survival (PFS) and overall survival (OS) in mRCC patients treated with immune checkpoint inhibitor(ICI). Other known prognostic clinical factors were also assessed, including prognostic scoring by MSKCC (Memorial Sloan Kettering) [9] and Heng criteria [7, 8] In addition race and age were evaluated to determine the role of these factors in the context of ICI therapy.

The primary objective of this study was to evaluate if NLR and response to duration of prior anti-VEGF therapy are predictors of clinical outcomes with ICI therapy in mRCC.

## Methods

This study was a retrospective chart review of patients who received or receiving PD-1 or PD-L1 inhibitor at Karmanos Cancer Institute(KCI). Regulatory approval was obtained from Wayne State University IRB. A retrospective chart review of mRCC patients at KCI, treated with any ICI therapy (PD-1/PD-L1 inhibitors) was conducted. Best response at any time during the therapy was collected.

The duration of anti-VEGF inhibitors was used as an objective surrogate reflecting response/efficacy with this therapy. If patients had received >1 prior anti-VEGF inhibitor, then the longest duration of each anti-VEGF inhibitor was utilized for coding. Data were collected on demographics, prognostic scoring (MSKCC and Heng), NLR pretherapy, and post 1 and 4 doses of ICI, duration of prior anti- VEGF therapy ≥6 months or less (as a surrogate for clinical benefit).

## Statistical methods

Baseline patient characteristics were summarized using count and percentage for categorical variables and median and range for continuous variables. Patient baseline characteristics were further compared between two groups (Pretherapy NLR <3 vs. Pretherapy NLR ≥3). Kruskal-Wallis tests were used to compare two groups for continuous variables and Chi-squared or Fisher’s exact tests for categorical variables. Progression-free survival (PFS) was calculated as the time from the date of PD1/PDL1 treatment to the date of progression or death from any cause. Patients who were alive without progression were considered censored at the date of last observation. Overall survival (OS) was calculated as the time from the date of PD1/PDL1 treatment to death from any cause. Patients who were alive were considered censored at the date of last observation. Kaplan-Meier estimates were used to summarize the distribution of PFS and OS. Univariable logistic regression models were fit to assess associations between the response of PD1/PDL1 (to progression disease and non-response with complete response and partial response and stable disease as a reference) and three prior chosen predictors (Heng prognostic score of favorable versus intermediate and poor risk, duration of prior anti-VEGF inhibitors with a cutoff of 6 months, and pretherapy NLR). Univariable cox proportional hazards regression models were fit to assess associations between three prior chosen predictors and survival benefit (PFS and OS). Multivariable logistic and Cox proportional hazards regression models were further fit to assess associations with the prior chosen covariates. The proportional hazard assumption was evaluated using Shoenfeld residuals and no violation was found. The median follow-up times were estimated using the reverse Kaplan-Meier method.

## Results

### Baseline characteristics

Forty-two patients were evaluated with median age of sixty-one years (range, 24-85). Nine patients (21%) were African American (AA), three (7%) were of Asian descent. Twenty-one patients (50%) were smokers**.** 33 (79%) of 42 patients were clear cell histology, 5 (12%) were papillary, 3 (7%) were sarcomatoid and 1 (2%) had medullary carcinoma. NLR median was 2.75 (range, 0.3-13.5). Pretherapy NLR < 3 and ≥3 was seen in nineteen (45%) and twenty-three (55%) patients respectively. Twenty-four (57%) and eighteen (43%) patients had prior anti-VEGF inhibitors for a duration of ≥6 months and <6 months, respectively. Twelve (29%), twenty-two (52%) and nine (8%) patients had favorable, intermediate and poor risk disease based on Heng criteria. Sixteen (38%) patients had bone metastases. Twenty- nine (69%) patients had received ≤1 prior therapy and thirteen (31%) had >1 prior therapy. Thirty-three patients (79%) received nivolumab. There was one patient that received a combination of PD1 and CTLA-4 inhibition (nivolumab and ipilimumab). There were eight patients treated with other ICI such as atezolizumab, pembrolizumab and avelumab (Table 1).

**Table 1:** Baseline Characteristics

	Pre NLR < 3 (*N* = 19)	Pre NLR ≥ 3 (*N* = 23)	All (*N* = 42)	*p*-value
Median, Age – median (range)	61 (45-85)	61 (24-82)	61 (24-85)	0.859
Race– no. (%)				0.339
Caucasian	14 (74)	16 (70)	30 (71)	
African-American	5 (26)	4 (17)	9 (21)	
Asian	0 (0)	3 (13)	3 (7)	
Histology– no. (%)				0.330
Clear Cell	17 (89)	16 (70)	33 (79)	
Non-Clear cell	2 (11)	9 (30)	9 (21)	
Smoking Status– no. (%)				>0.99
No	10 (53)	11 (48)	21 (50)	
Yes	9 (47)	12 (52)	21 (50)	
Number of Prior anti-VEGF Therapies– no. (%)				0.093
≤1	16 (84)	13 (57)	29 (69)	
> 1	3 (16)	10 (43)	13 (31)	
Duration of prior anti-VEGF Therapies– no. (%)				>0.99
< 6 Months	8 (42)	10 (43)	18 (43)	
≥ 6 Months	11 (58)	13 (57)	24 (57)	
NLR at Day 15– no. (%)				*<0.001*
< 3	15 (79)	2 (9)	17 (40)	
≥ 3	4 (21)	20 (87)	24 (57)	
NLR at Cycle 3– no. (%)^a^				*0.001*
< 3	13 (68)	3 (13)	16 (38)	
≥ 3	4 (21)	16 (70)	20 (48)	
Types of anti-VEGF Therapies – no. (%)				
Pazopanib	10 (34)	10 (77)	20 (48)	*0.019*
Sunitinib	6 (21)	9 (69)	15 (36)	*0.005*
Axitinib	0 (0)	9 (69)	9 (21)	*<0.001*
Sorafenib	0 (0)	6 (46)	6 (14)	*<0.001*
Bevacizumab	2 (7)	5 (38)	7 (17)	*0.021*
Everolimus	2 (7)	5 (38)	7 (17)	*0.021*
IL2	10 (34)	10 (77)	20 (48)	*0.019*
Histology – no. (%)				0.330
Clear	17 (89)	16 (70)	33 (79)	
Clear Cell W/ Sarcamatoid Features	0 (0)	3 (13)	3 (7)	
Clear Cell W/ Papillary Features	2 (1)	3 (13)	5 (12)	
Medullary	0 (0)	1 (4)	1 (2)	
Heng Prognostic Score– no. (%)				0.509
Low	4 (21)	8 (35)	12 (29)	
Intermediate	10 (53)	12 (52)	22 (52)	
High	5 (26)	3 (13)	8 (19)	
MSKCC Prognostic Score– no. (%)				0.317
Low	4 (21)	9 (39)	13 (31)	
Intermediate	15 (79)	14 (61)	29 (69)	
Type of Immune Checkpoint Inhibitor Therapy– no. (%)				0.220
Nivolumab	19 (66)	10 (77)	29 (69)	
Nivolumab plus Nexavar	0 (0)	1 (8)	1 (2)	
Nivolumab plus Votrient	1 (3)	2 (15)	3 (7)	
Nivolumab + Ipilimumab	1 (3)	0 (0)	1 (2)	
Ipilimumab	0 (0)	0 (0)	0 (0)	
Avelumab	1 (3)	0 (0)	1 (2)	
Pembrolizumab and Axitinib	5 (17)	0 (0)	5 (12)	
Atezolizumab and Avastin	2 (7)	0 (0)	2 (5)	

^a^Data are not available for 6 patients

### NLR

NLR was evaluated at three distinct time points; pretherapy, after 1st dose and after 4th dose via univariable and multivariable analyses. Univariable response rate was evaluated (NLR ≥3 vs. <3, <3 as reference category), and showed that patients with NLR ≥3 had a lower likelihood of response(response rate [RR]: 52% vs. 74%) compared to those with NLR <3 for pretherapy NLR, after 1st dose and after 4th dose (pretherapy: OR 2.57, 95% CI, 0.72 to 10.16; *p* = 0.158; after 1st dose: OR 2.75; 95% CI, 1.24 to 5.17; p = 0.15; after 4th dose: OR 2.33; 95% CI, 1.39 to 6.57; *p* = 0.286; Table 2 and Additional file 1: Table S1).

**Table 2:** Univariable and multivariable logistic and Cox regression analyses of risk factors associated with RR, PFS, and OS

	RR^*^				PFS^#^				OS^$^			
	Univariable analysis		Multivariable analysis		Univariable analysis		Multivariable analysis		Univariable analysis		Multivariable analysis	
	OR (95% CI)	*p-value*	OR (95% CI)	*p-value*	HR (95% CI)	*p-value*	HR (95% CI)	*p-value*	HR (95% CI)	*p-value*	HR (95% CI)	*p-value*
Heng Prognostic Score												
Low	Reference		Reference		Reference		Reference		Reference		Reference	
Int/High^^^	0.500 (0.124,1.973)	0.319	0.553 (0.130,2.322)	0.414	0.779 (0.379,1.6)	0.496	0.892 (0.433,1.837)	0.757	0.514 (0.182,1.451)	0.201	0.559 (0.197,1.585)	0.274
Duration of prior anti-VEGF Therapies												
< 6 Months	Reference		Reference		Reference		Reference		Reference		Reference	
≥ 6 Months	2.200 (0.613,8.678)	0.237	2.362 (0.627,9.979)	0.216	2.015 (1.046,3.883)	*0.048*	2.288 (1.094,4.785)	*0.028*	2.869 (0.9,9.141)	0.063	2.913 (0.904,9.388)	0.073
Pretherapy NLR												
< 3	Reference		Reference		Reference		Reference		Reference		Reference	
≥ 3	2.567 (0.717,10.16)	0.158	2.538 (0.674,10.57)	0.178	2.67 (1.343,5.308)	*0.004*	2.937 (1.444,5.972)	*0.003*	3.977 (1.227,12.889)	*0.014*	4.01 (1.189,13.524)	*0.025*

^^^, Intermediate and high; ^*^, PD1/PDL1 response rate to progression disease and non-response; ^#^, progression-free survival; ^$^, overall survival

For NLR pretherapy, after 1st dose and after 4th dose (NLR ≥3 vs. <3, <3 as reference category), univariable PFS analyses showed that patients with NLR ≥3 have a higher risk of progression than those with NLR <3 (pretherapy: HR, 2.670; 95% CI, 1.34 to 5.31; *p* = 0.004; after 1st dose: HR, 2.532; 95% CI, 1.24 to 5.17; *p* = 0.009; after 4th dose: HR, 3.017; 95% CI, 1.39 to 6.57; *p* = 0.004; Table 2 and Additional file 1: Table S1).

**Fig. 1:**
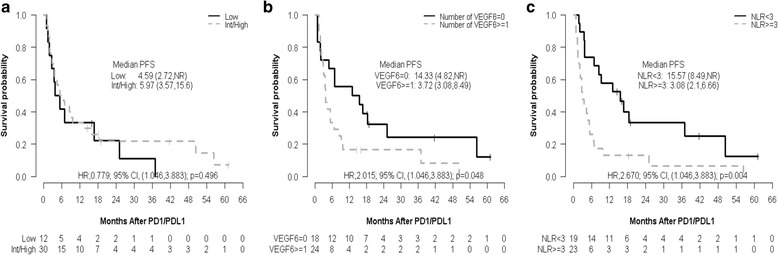
Progression—free survival (PFS) estimates by (**a**) Heng prognostic score, (**b**) the duration of prior anti—VEGF inhibitors ≥6 months, and (**c**) pretherapy NLR. The median follow---up times are (**a**) NR (16.1,NR) months for ‘Low’ and 41.8 (19.0, NR) months for ‘Int/High’, (**b**) 41.8 (19.0,NR) months for ‘VEGF6 = 0’ and 16.1 (14.7,NR) months for ‘VEGF6 ≥ 1’, and (**c**) 41.8 (19.0,NR) months for ‘NLR < 3’ and NR (18.4,NR) months for ‘NLR ≥ 3’

**Fig. 2:**
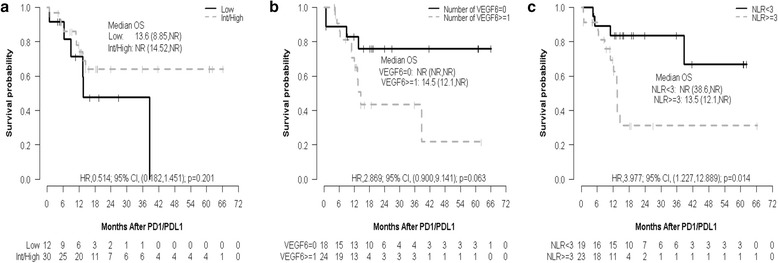
Overall survival (OS) estimates by (**a**) Heng prognostic score, (**b**) the duration of prior anti—VEGF inhibitors ≥6 months, and (**c**) pretherapy NLR. The median follow---up times are (**a**) 19.7 (10.9,NR) months for ‘Low’ and 18.9 (14.7, 41.8) months for ‘Int/High’, (**b**) 19.7 (18.4,61.2) months for ‘VEGF6 = 0’ and 14.7 (12.7,NR) months for ‘VEGF6 ≥ 1’, and (**c**) 19.7 (16.1,61.2) months for ‘NLR < 3’ and 18.4 (12.0,NR) months for ‘NLR ≥ 3’

**Fig. 3:**
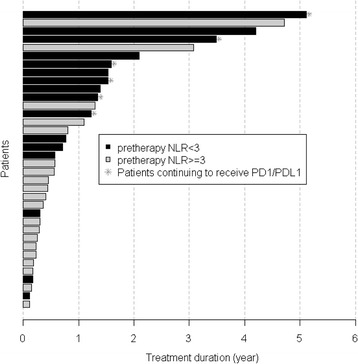
The duration of PD1/PDL1 treatment in year

Multivariable PFS analyses showed that patients with pretherapy NLR ≥3 have 2.937 higher risk of progression than those with NLR <3 (HR, 2.937; 95% CI,1.44 to5.97; *p* = 0.003). Similarly, univariable OS analyses showed that patients with NLR ≥3 have a higher risk of death than those with NLR <3 (pretherapy: HR,3.977; 95% CI, 1.23 to 12.89; *p* = 0.014; after 1st dose: HR,4.856; 95% CI, 1.31 to 18.01; *p* = 0.008; after 4th dose: HR,12.935; 95% CI, 1.64 to 101.94; *p* = 0.001; Table 2 and Additional file 1: Table S1). Pretherapy NLR <3 demonstrated a longer PFS and OS as compared to NLR ≥ 3 (median PFS: 15.57 vs. 3.08 months; p = 0.004; median OS: not reached vs. 13.50 months, p = 0.01) (Figs. 1 and 2). The duration of therapy in the groups is depicted as a swimmers plot [Fig. 3].

## Prior anti-Vegf therapy

The duration of prior anti-VEGF therapy was considered to be an adequate surrogate for clinical benefit with prior therapy. The cutoff of 6 months was chosen as an adequate time period to establish tolerability and clinical benefit with anti-VEGF therapy. Univariable PFS and OS analyses showed that patients with ≥6 months of anti-VEGF therapy have a significantly higher risk of progression and a marginally higher risk of death than those with anti-VEGF therapy duration of <6 months (PFS: HR, 2.015; 95% CI, 1.05 to 3.88; *p* = 0.048; OS: HR, 2.869; 95% CI, 0.90 to 9.14; *p* = 0.063). This factor remained a statistically significant predictor of outcome in the multivariable PFS analysis(HR, 2.288; 95% CI, 1.09 to 4.79; *p* = 0.028) and marginally significant in the multivariable OS analysis (HR, 2.913; 95% CI, 0.90 to 9.39; *p* = 0.073). Univariable analysis was done on response rate correlating with duration of anti-VEGF therapy and patients with duration ≥6 months of anti-VEGF therapy showed a decreased likelihood of response to ICI therapy (OR 2.200, 95%CI 0.61 to 8.68, *p*-value 0.237) (Table 2).

We also evaluated effect of response to prior anti-VEGF therapy in patients who were on multiple anti-VEGF therapies. We found that those with >1 anti-VEGF therapy tended to have a higher risk of progression on ICI therapy, but the p-value was not statistically significant (HR, 1.668; 95% CI, 0.83 to 3.37; *p* = 0.167), and to have a significantly higher risk of death than those with ≤1 prior anti-VEGF therapy (HR, 3.424; 95% CI, 1.23 to 9.57; *p* = 0.021) based on univariable analysis (Additional file 1: Table S1).

The patients with duration of prior anti-VEGF therapy <6 months or lack of clinical benefit with anti-VEGF therapy, were more likely to benefit from ICI therapy compared to those receiving therapy for ≥6 months (median PFS: 14.33 and 3.72 months for <6 and ≥6 months, respectively; *p* = 0.048) (Table 2, Figs. 1 and 2, Additional file 1: Figure S1).

## Race

Univariable analyses for PFS and OS showed that non-Caucasians (9 African American, 2 Middle Eastern and 1 Asian) had a higher risk of progression and death compared to Caucasians (PFS: HR, 3.362; 95% CI, 1.55 to 7.31; *p* = 0.004; OS: HR, 8.666; 95% CI, 2.87 to 26.16; *p* < 0.001) (Additional file 1: Table S1).

Univariable analyses for RR showed that non-Caucasians had a significantly decreased response rate to ICI therapy (RR: 25% vs. 77%) than Caucasians (OR 9.857, 95% CI 2.27 to 54.83, p = 0.004) (Additional file 1: Table S1).

### Smoking

Smoking status was not found to be significant in affecting either PFS or OS. Univariable smoking status (yes vs. no, no as reference category) for PFS is HR 1.510 (95% CI, 0.78 to 2.92; *p* = 0.221) and for OS HR 1.305 (95% CI, 0.47 to 3.6; *p* = 0.608). Univariable analysis of response rate showed an OR of 2.273 (95% CI 0.65 to 8.54, *p* = 0.208) (Additional file 1: Table S1).

### Other prognostic assessment

Heng prognostic scoring was also assessed and is categorized it into favorable, intermediate and high categories, however for statistical purposes intermediate and high were combined. Univariable PFS and OS analyses showed intermediate/high vs. low with HR 0.779(95% CI, 0.38 to 1.6) and HR 0.514(95% CI, 0.18 to 1.45) with the *p*-value of 0.496 for PFS and 0.201 for OS (Table 2). The response rate was evaluated using univariable analysis and showed an OR 0.500 (95% CI 0.124 to 1.973, *p* = 0.319).

MSKCC prognostic scoring was also evaluated and patients were subdivided into low, intermediate and high, however, there were no patients in high-risk category (low as reference category). With univariable analysis, the intermediate versus low risk had a HR of 0.764(95% CI, 0.38 to 1.55; *p* = 0.462) for PFS and HR of 0.428(95% CI, 0.15 to 1.19; *p* = 0.111) for OS. The response rate was analyzed with univariable analysis and showed an OR 0.614 (95% CI 0.16 to 2.37, *p* = 0.473) (Additional file 1: Figure S1, Tables S2, S3, S4 and S5). The *p*-values did not reach statistical significance for prediction of clinical outcomes with ICI therapy within the Heng or MSKCC prognostic categories.

## Discussion

Therapy with ICI is rapidly establishing efficacy, not only in RCC but also in various other tumor types such as urothelial, lung, Merkel cell cancers and melanoma. Finding a simple universally applicable predictor of response would represent an invaluable clinical tool for treatment decisions. In this study, NLR and duration of prior anti-VEGF therapy emerge as significant biomarkers prognostic of clinical outcomes with ICI therapies in mRCC. NLR is a simple clinical tool that can be utilized without any additional cost, or sample collection. To our knowledge this is the first report utilizing NLR and duration of prior anti-VEGF therapy as prognostic biomarkers in the context of ICI therapy in mRCC.

In mRCC there is a pressing need for biomarkers especially as PD-1 or PDL-1 expression testing failed to reveal an association with response or clinical outcomes. Front line trials are ongoing to explore the role of ICI therapies and if NLR is validated it may allow a rational method of making therapeutic choices in RCC and developing pathways and sequences of therapies. Our study found that pretherapy NLR <3 is a statistically significant predictor of response, and improved PFS and OS with ICI therapy in RCC. Baum et al11] showed decreased OS with a cutoff of preoperative NLR ≥4 in cytoreductive nephrectomy patients with mRCC. The NLR cutoff of 3 that is primarily used in this study, was adopted from a meta-analysis conducted by Hu et al. incorporating 15 studies evaluating NLR as a prognostic factor in RCC. We evaluated our database with NLR 4 cutoff and found that pretherapy NLR ≥ 4 also was associated with worse PFS with ICI in RCC with a HR of 2.546 (95% CI, 1.25 to 5.19; *p* = 0.01) in univariable analysis but non-significant association with OS (HR, 2.19; 95% CI, 0.77 to 6.22; *p* = 0.14). We also noted that the duration of prior anti- VEGF therapy of 6 months or longer, had a lower possibility of benefit from ICI. It would appear that patients with RCC that are sensitive to anti-VEGF TKI therapy are unlikely to respond to single agent ICI therapy.

Despite the intriguing findings, this study has multiple limitations. Extraneous factors that may affect NLR such as steroid therapy have to be taken into consideration. The small sample size, retrospective nature of the study, the assumptions of all ICI having similar potential of efficacy in mRCC and lack of centralized review of scans for response and progression represent the main ones. However 52% of the patients were treated on clinical trials with uniform criteria to assess progression and toxicity and this may overcome the latter limitation, as well as those treated off clinical trial were evaluated by the same physician and scans were done at least every 12 weeks. In addition, the single institution nature of the trial also avoids the heterogeneity in patient assessment and treatment patterns.

The hypotheses generated by this study are worthy of further investigations given the large magnitude of differences and statistical significance findings in a multivariable analysis setting. The current paucity of predictive biomarker availability for therapeutic decision making and the recent growth in therapeutic choices in mRCC further underline the importance of exploring these clinical criteria. Optimizing currently available therapeutic options in mRCC is a dire need to streamline cost, and optimize risk benefit ratio in the management of mRCC patients. Further validation of this observation is required in a larger sample size with multi-institutional collaboration, which will occur in the future.

Despite the limitations a few observations are noteworthy from the current study. A higher NLR is associated with worse prognosis in the setting of ICI therapy, which is consistent with other studies in the literature describing the impact of NLR on clinical outcomes in the setting of sunitinib therapy [13, 14].

Due to the retrospective nature of the study no tumor tissue or peripheral blood samples were available. However future prospective testing will need to incorporate a variety of immunologic predictive biomarkers such as serum lactate dehydrogenase and C reactive protein and tumor PDL-1 status, mutation load, T cell subsets and changes in and presence of tumor infiltrating lymphocytes [15]. However, preliminary clinical correlation indicates a lack of predictive capacity for immune markers in mRCC. In the Checkmate 025 study [1], however PDL-1 status did not correlate with clinical outcomes in RCC patients treated with nivolumab.

Our study results indicate that using NLR and prior VEGF therapy duration would potentially enable us to select patients who are more likely to benefit from ICI therapy in the second line setting. As therapeutic arsenal continues to expand and more therapies are approved in RCC, the sequencing of these therapies will continue to present a critical challenge. Future investigations with a larger sample size and with a control arm are warranted to validate the predictive capacity of the above biomarkers.

## Conclusions

In conclusion, utilization of universally available and easily applicable biomarkers such as NLR and duration of prior anti-VEGF therapy, would allow us to rationally determine appropriate therapeutic sequence and optimize outcomes and cost of therapy in mRCC.

## Additional files

Additional file 1: Table S1. Univariable logistic and Cox regression analyses for RR, PFS, and OS. **Table S2**. Univariable and multivariable logistic and Cox regression analyses of risk factors associated with RR, PFS, and OS. Note ‘Pretherapy NLR’ is grouped by its median of 3.2. **Table S3**. Univariable and multivariable logistic and Cox regression analyses of risk factors associated with RR, PFS, and OS. Note ‘Pretherapy NLR’ is considered as a continuous variable. **Table S4**. Univariable and multivariable Cox regression analyses of risk factors associated with PFS. Note that two variables ‘Duration of prior anti-VEGF Therapies’ and ‘Pretherapy NLR (with the cutoff value of 3)’ are combined. **Table S5**. Univariable and multivariable Cox regression analyses of risk factors associated with PFS. Note that two variables ‘Duration of prior anti-VEGF Therapies’ and ‘Pretherapy NLR (with the cutoff value of 3.2 [median])’ are combined. **Figure S1**. The boxplot of pretherapy NLR by the duration of prior anti-VEGF therapies. The *p*-value is calculated using the Kruskal. (DOCX 38 kb)

## Abbreviations

ICI: Immune Checkpoint Inhibitors
KCI: Karmanos Cancer Institute
mRCC: Metastatic Renal Cell Carcinoma
OS: Overall Survival
PFS: Progression Free Survival
RR: Response Rate
