# Supplementary material for: Neutrophil lymphocyte ratio and duration of prior anti-angiogenic therapy as biomarkers in metastatic RCC receiving immune checkpoint inhibitor therapy
Source: J Immunother Cancer. 2017 Oct 17;5:82. doi: 10.1186/s40425-017-0287-5 (PMC5646127; doi:10.1186/s40425-017-0287-5)
Supplement: Supplementary file 1 — Univariable logistic and Cox regression analyses for RR, PFS, and OS. Table S2. Univariable and multivariable logistic and Cox regression analyses of risk factors associated with RR, PFS, and OS. Note ‘Pretherapy NLR’ is grouped by its median of 3.2. Table S3. Univariable and multivariable logistic and Cox regression analyses of risk factors associated with RR, PFS, and OS. Note ‘Pretherapy NLR’ is considered as a continuous variable. Table S4. Univariable and multivariable Cox regression analyses of risk factors associated with PFS. Note that two variables ‘Duration of prior anti-VEGF Therapies’ and ‘Pretherapy NLR (with the cutoff value of 3)’ are combined. Table S5. Univariable and multivariable Cox regression analyses of risk factors associated with PFS. Note that two variables ‘Duration of prior anti-VEGF Therapies’ and ‘Pretherapy NLR (with the cutoff value of 3.2 [median])’ are combined. Figure S1. The boxplot of pretherapy NLR by the duration of prior anti-VEGF therapies. The p-value is calculated using the Kruskal. (DOCX 38 kb) [file 40425_2017_287_MOESM1_ESM.docx]

**Supplementary Information**

**Table S1. Univariable logistic and Cox regression analyses for RR, PFS, and OS.**

|  | **RR** |  | **PFS** |  | **OS** |  |
| --- | --- | --- | --- | --- | --- | --- |
|  | **OR (95% CI)** | **p-value** | **HR (95% CI)** | **p-value** | **HR (95% CI)** | **p-value** |
| **Race** |  | ***0.004*** |  | ***0.004*** |  | ***<0.001*** |
| **Caucasian** | Reference |  | Reference |  | Reference |  |
| **Others^*^** | 9.857 (2.273,54.832) |  | 3.362 (1.546,7.312) |  | 8.666 (2.871,26.159) |  |
| **Smoking Status** |  | 0.208 |  | 0.221 |  | 0.608 |
| **No** | Reference |  | Reference |  | Reference |  |
| **Yes** | 2.273 (0.647,8.540) |  | 1.51 (0.78,2.926) |  | 1.305 (0.471,3.610) |  |
| **Number of Prior anti-VEGF Therapies** |  | 0.165 |  | 0.167 |  | ***0.021*** |
| **≤ 1** | Reference |  | Reference |  | Reference |  |
| **> 1** | 2.593 (0.680,10.357) |  | 1.668 (0.825,3.374) |  | 3.424 (1.226,9.566) |  |
| **Number of Prior anti-VEGF Therapies (Continuous)** | 1.412 (0.898,2.333) | 0.146 | 1.135 (0-.901,1.430) | 0.282 | 1.429 (1.018,2.006) | ***0.039*** |
| **NLR at Day 15** |  | 0.150 |  | ***0.009*** |  | ***0.008*** |
| **< 3** | Reference |  | Reference |  | Reference |  |
| **≥ 3** | 2.750 (0.728,12.047) |  | 2.532 (1.241,5.166) |  | 4.856 (1.309,18.011) |  |
| **NLR at Cycle 3** |  | 0.286 |  | ***0.004*** |  | ***0.001*** |
| **< 3** | Reference |  | Reference |  | Reference |  |
| **≥ 3** | 2.333 (0.521,12.728) |  | 3.017 (1.386,6.569) |  | 12.935 (1.641,101.941) |  |
| **MSKCC Prognostic Score** |  | 0.473 |  | 0.462 |  | 0.111 |
| **Low** | Reference |  | Reference |  | Reference |  |
| **Intermediate** | 0.614 (0.159,2.372) |  | 0.764 (0.378,1.548) |  | 0.428 (0.154,1.187) |  |

*, Others include African-American and Asian.

**Table S2. Univariable and multivariable logistic and Cox regression analyses of risk factors associated with RR, PFS, and OS. Note ‘Pretherapy NLR’ is grouped by its median of 3.2.**

|  | **RR^*^** | | | | **PFS^#^** | | | | **OS^$^** | | | |
| --- | --- | --- | --- | --- | --- | --- | --- | --- | --- | --- | --- | --- |
|  | **Univariable analysis** | | **Multivariable analysis** | | **Univariable analysis** | | **Multivariable analysis** | | **Univariable analysis** | | **Multivariable analysis** | |
|  | **OR (95% CI)** | **p-value** | **OR (95% CI)** | **p-value** | **HR (95% CI)** | **p-value** | **HR (95% CI)** | **p-value** | **HR (95% CI)** | **p-value** | **HR (95% CI)** | **p-value** |
| **Heng Prognostic Score** |  |  |  |  |  |  |  |  |  |  |  |  |
| **Low** | Reference |  | Reference |  | Reference |  | Reference |  | Reference |  | Reference |  |
| **Int/High^^^** | 0.500 (0.124,1.973) | 0.319 | 0.551 (0.130,2.303) | 0.409 | 0.779 (0.379,1.6) | 0.496 | 0.911 (0.442,1.876) | 0.799 | 0.514 (0.182,1.451) | 0.201 | 0.591 (0.209,1.671) | 0.321 |
| **Duration of prior anti-VEGF Therapies** |  |  |  |  |  |  |  |  |  |  |  |  |
| **<6 Months** | Reference |  | Reference |  | Reference |  | Reference |  | Reference |  | Reference |  |
| **≥6 Months** | 2.200 (0.613,8.678) | 0.237 | 2.363 (0.636,9.841) | 0.212 | 2.015 (1.046,3.883) | ***0.048*** | 2.298 (1.102,4.792) | ***0.027*** | 2.869 (0.9,9.141) | 0.063 | 2.978 (0.929,9.55) | 0.066 |
| **Pretherapy NLR** |  |  |  |  |  |  |  |  |  |  |  |  |
| **<3.2** | Reference |  | Reference |  | Reference |  | Reference |  | Reference |  | Reference |  |
| **≥3.2** | 1.944 (0.554,7.257) | 0.306 | 1.926 (0.516,7.653) | 0.335 | 2.466 (1.253,4.853) | ***0.009*** | 2.731 (1.358,5.491) | ***0.005*** | 3.121 (1.039,9.37) | ***0.042*** | 3.156 (1.02,9.763) | ***0.046*** |

^^^, Intermediate and high; ^*^, PD1/PDL1 response rate to progression disease and non-response; ^#^, progression-free survival; ^$^, overall survival.

**Table S3. Univariable and multivariable logistic and Cox regression analyses of risk factors associated with RR, PFS, and OS. Note ‘Pretherapy NLR’ is considered as a continuous variable.**

|  | **RR^*^** | | | | **PFS^#^** | | | | **OS^$^** | | | |
| --- | --- | --- | --- | --- | --- | --- | --- | --- | --- | --- | --- | --- |
|  | **Univariable analysis** | | **Multivariable analysis** | | **Univariable analysis** | | **Multivariable analysis** | | **Univariable analysis** | | **Multivariable analysis** | |
|  | **OR (95% CI)** | **p-value** | **OR (95% CI)** | **p-value** | **HR (95% CI)** | **p-value** | **HR (95% CI)** | **p-value** | **HR (95% CI)** | **p-value** | **HR (95% CI)** | **p-value** |
| **Heng Prognostic Score** |  |  |  |  |  |  |  |  |  |  |  |  |
| **Low** | Reference |  | Reference |  | Reference |  | Reference |  | Reference |  | Reference |  |
| **Int/High^^^** | 0.500 (0.124,1.973) | 0.319 | 0.468 (0.110,1.919) | 0.290 | 0.779 (0.379,1.6) | 0.496 | 0.656 (0.308,1.398) | 0.274 | 0.514 (0.182,1.451) | 0.201 | 0.533 (0.187,1.513) | 0.237 |
| **Duration of prior anti-VEGF Therapies** |  |  |  |  |  |  |  |  |  |  |  |  |
| **<6 Months** | Reference |  | Reference |  | Reference |  | Reference |  | Reference |  | Reference |  |
| **≥6 Months** | 2.200 (0.613,8.678) | 0.237 | 2.206 (0.600,8.947) | 0.244 | 2.015 (1.046,3.883) | ***0.048*** | 1.885 (0.908,3.913) | 0.089 | 2.869 (0.9,9.141) | 0.063 | 2.541 (0.781,8.271) | 0.122 |
| **Pretherapy NLR** | 1.102 (0.848,1.449) | 1.449 | 1.115 (0.851,1.479) | 0.423 | 1.328 (1.146,1.538) | ***<0.001*** | 1.337 (1.149,1.556) | ***<0.001*** | 1.123 (0.926,1.361) | 0.237 | 1.091 (0.901,1.321) | 0.371 |

^^^, Intermediate and high; ^*^, PD1/PDL1 response rate to progression disease and non-response; ^#^, progression-free survival; ^$^, overall survival.

**Table S4. Univariable and multivariable Cox regression analyses of risk factors associated with PFS. Note that two variables ‘Duration of prior anti-VEGF Therapies’ and ‘Pretherapy NLR (with the cutoff value of 3)’ are combined.**

|  | **PFS^*^** | | | |
| --- | --- | --- | --- | --- |
|  | **Univariable analysis** | | **Multivariable analysis** | |
|  | **HR (95% CI)** | **p-value** | **HR (95% CI)** | **p-value** |
| **Heng Prognostic Score** |  |  |  |  |
| **Low** | Reference |  | Reference |  |
| **Int/High^^^** | 0.779 (0.379,1.6) | 0.496 | 0.886 (0.424,1.851) | 0.748 |
| **Duration of prior anti-VEGF Therapies & Pretherapy NLR** |  |  |  |  |
| **<6 Months & <3** | Reference |  | Reference |  |
| **<6 Months & ≥3** | 2.904 (0.967,8.724) | 0.057 | 2.819 (0.924,8.600) | 0.069 |
| **≥6 Months & <3** | 2.266 (0.75,6.845) | 0.147 | 2.198 (0.716,6.748) | 0.169 |
| **≥6 Months & ≥3** | 6.733 (2.194,20.659) | ***0.001*** | 6.636 (2.153,20.457) | ***0.001*** |

^^^, Intermediate and high; ^*^, PD1/PDL1 response rate to progression disease and non-response; ^#^, progression-free survival.

**Table S5. Univariable and multivariable Cox regression analyses of risk factors associated with PFS. Note that two variables ‘Duration of prior anti-VEGF Therapies’ and ‘Pretherapy NLR (with the cutoff value of 3.2 [median])’ are combined.**

|  | **PFS^*^** | | | |
| --- | --- | --- | --- | --- |
|  | **Univariable analysis** | | **Multivariable analysis** | |
|  | **HR (95% CI)** | **p-value** | **HR (95% CI)** | **p-value** |
| **Heng Prognostic Score** |  |  |  |  |
| **Low** | Reference |  | Reference |  |
| **Int/High^^^** | 0.779 (0.379,1.6) | 0.496 | 0.916 (0.439,1.908) | 0.814 |
| **Duration of prior anti-VEGF Therapies & Pretherapy NLR** |  |  |  |  |
| **<6 Months & <3** | Reference |  | Reference |  |
| **<6 Months & ≥3** | 2.893 (0.963,8.689) | 0.058 | 2.831 (0.928,8.633) | 0.067 |
| **≥6 Months & <3** | 2.427 (0.818,7.196) | 0.110 | 2.376 (0.79,7.149) | 0.123 |
| **≥6 Months & ≥3** | 6.422 (2.085,19.777) | ***0.001*** | 6.341 (2.047,19.641) | ***0.001*** |

^^^, Intermediate and high; ^*^, PD1/PDL1 response rate to progression disease and non-response; ^#^, progression-free survival.


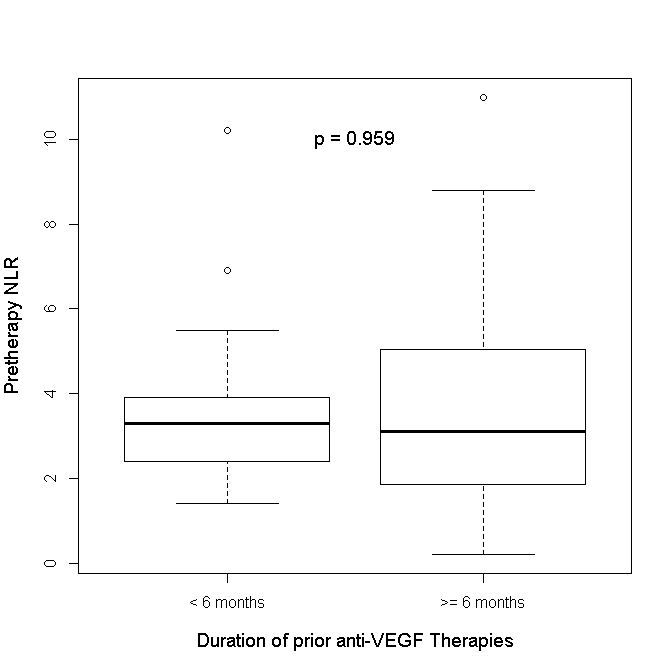


**Figure S1. The boxplot of pretherapy NLR by the duration of prior anti-VEGF therapies**. The p-value is calculated using the Kruskal
